# Comparative study of double and single plate fixation for distal femur fractures

**DOI:** 10.6026/973206300220157

**Published:** 2026-01-31

**Authors:** Arvind Ambedkar, Alka Ambedkar, Darshita Sharma, Sachin Parmar

**Affiliations:** 1 Department of Orthopaedic Specialist, District Hospital Shahdol, Madhya Pradesh, India; 2 Department of Pathology, BMGMC Shahdol, Madhya Pradesh, India; 3 Department of Pathology, GMC, Singrauli, Madhya Pradesh, India; 4 Department of Community Medicine, V.K.S. Government Medical College, Neemuch, Madhya Pradesh, India

**Keywords:** Distal femur fracture, double LCP fixation, fracture union, functional outcome

## Abstract

Distal femur fractures account for less than 1% of all fractures and approximately 3-6% of femoral fractures. Hence, 30 patients underwent double plating using a distal femur locking compression plate (LCP) at the J.A. Group of Hospitals, Gwalior. Radiographs and the Knee Society Score were used to evaluate outcomes after dual plating through lateral and medial approaches. Overall, 89.99% of patients achieved excellent-to-good results, 30% developed minor complications, and most fractures united by 20-22 weeks. In complex distal femur fractures, double distal femur LCP fixation appears to provide stable fixation, reliable union, and satisfactory functional recovery.

## Background:

Comminuted distal femur fracture is a clinical problem and consists about 3-6 percent of all femur fractures and less than 1 percent of all fractures [1]. These fractures are found to be bimodally distributed with numbers peaking on young men after high energy trauma and the elderly women having low energy osteoporotic fractures [2]. The prevalence of distal femur fracture is 8.7 events per 100,000 individuals per year, and it significantly increases after age 60 years, especially in females (66.6 percent) as opposed to males (33.4 percent). The average age when fracture happens is 62.2 years there is a difference of 44.0 years and 71.6 years between men and women [3]. Low-energy injuries are 97 percent; about 61 percent of them are caused by standing height fall [4]. These fractures are classified using the AO/OTA classification in which they are described as extra-articular (Type A), partial-articular (Type B), and complete articular (Type C) [5]. Intra-articular fractures represent 38.6 percent and complex comminuted intra-articular Type C3 fractures are particularly challenging in terms of therapeutic management, as there is tremendous comminution across the metaphysis and articular surfaces are involved. Treatment of these fractures is to obtain anatomical reduction, stable fixation, early mobilization and functional recovery with the minimal complications [1]. The recent addition of Locking Compression Plates (LCP) to the options in treating distal femur fractures has demonstrated not only biomechanical benefits, but also unique properties of their design [6]. LCP is a single beam structure with fixation strength being representable by the number of all screws in bone interfaces instead of the pull-out resistance of each of the screws [7]. There is flexible stabilization in this mechanism to close the gap with maintenance of periosteal blood supply thus facilitating biological healing. Compared with standard screws, locking screw design produces a higher pull-out strength; thus, of interest in osteoporotic bone [8, 9].

Nevertheless, despite the mentioned advantages, single lateral plate fixation proves to have considerable limitations in complex plans of the fracture [10]. The use of technical issues and mechanical locking compression plate failure of, distal femoral locking plate require revision surgery or 12.8 percent of the total distal femoral locking process and the most widespread problems are fixation failure, deep infection and malunion [11]. The most prevalent type of complication that occurs is the nonunion rate with 14.8%. The failure modes are also varus collapse, implant failure, and loss of reduction [12]. The reports of the comparative studies carried out indicate that 18% of a metalwork fails and that there is a higher rate of complications in the single lateral plating particularly in patients with poor bone quality [13]. To overcome these defects, dual plating (double LCP fixation) has been developed that results in orthogonal fixation plates giving it increased biomechanical advantages. Biomechanical analysis shows that dual plate construct has almost twice axial and rotational stiffness and displacement and less number of failure incidences than single lateral plating [14]. This procedure transforms cantilever fixation to on-axis fixation and it supports medial column and prohibits varus collapse. Meta-analysis demonstrates that the sole use of a plate provides more mobility of knee joint in 6 months after the surgery, the overall complication rates are smaller (7.32 percent versus 17 percent) and less malunion occurs in comparison with the isolated use of a plate [15, 16]. The indications of the dual plating are comminuted distal femur (AO type A2, A3, C1, C2, and C3), medial supracondylar bone loss more than 2 cm, low trans-condylar-bicondylar fractures, periprosthetic distal femur and nonunion of failed single lateral plate. Systematic review shows that dual plating has nonunion rate of as high as 12.5 percent compared to the individual lateral plate nonunion rate which approximates 14 percent and the fracture healing is faster than the single plates where surgery takes longer. There is a current evidence stating that in complex, comminuted distal femur fractures, single lateral plate plating might not offer enough stabilization and could not be considered as offering sufficient stability and hence resulting in avoidance of complications, at which point dual plating should be contemplated [17, 18]. Therefore, it is of interest to report the comparative study of double and single plate fixation for distal femur fractures.

## Methods and Materials:

## Study design and setting:

The study belongs to the prospective observational cohorts, which took place in the department of orthopaedics and trauma centre of J.A. Group of Hospitals, Gwalior, and Madhya Pradesh, India. This study began in October of 2016 and the enrollment of patients started with the attendance of the routine visits at the outpatient department. All the procedures have been carried out following ethical reports of the institutional research committee and the Helsinki declaration of 1964 as well as the modifications made to it later on.

## The population and sample size of the study:

The enrollment was made prospectively and 30 patients with comminuted distal femur fractures were enrolled in the study till the end of the study period. The determination of sample size used feasibility and projected recruitment rate of the outpatient department. Follow-up of patients was done at least 6-8 months after intervention period to determine functional and surgical outcomes.

## Inclusion and Exclusion criteria:

## Inclusion criteria:

[1] Patients in the age group of 18-60 years who had complete clinical records

[2] Intra-articular distal femoral comminuted type C according to the AO classification

[3] Medically and surgically fit patients that can be taken on operative intervention

[4] Ability to sign informed written consent

[5] No record of metastatic disease

## Exclusion criteria:

[1] The refusal of the patient to sign informed consent

[2] Under age

[3] Suspicious pathological fractures

[4] High-level radiological correlation of osteoarthritis or rheumatoid arthritis of the affected limb

[5] Bedridden or other patients with little mobility (moved between bed and chair only)

[6] AO classification type A and B fractures

## Pre-operative planning and pre-operative assessment:

All the patients were thoroughly assessed as per their general status on admission and specific focus was done upon orthopaedic associated harm and systemic injury. Data gathering was done through pre-determined data collection forms both directly or through the medical records of the patients. Before the intervention, all the patients were counseled on modes of treatment and gave legitimate written informed consent. Pre-operative tests were full blood count with haemoglobin count, random blood sugar, blood urea, serum creatinine, HIV serology (consenting patients), hepatitis b surface antigen, chest X-ray, electrocardiogram and echocardiogram as required. Medium-length X-rays of the affected limb in the position of anteroposterior and lateral projections of the surrounding joints were taken. The AO/OTA classification system was used to describe patterns of fractures. Before the operative intervention was carried out, medical, surgical and anesthetic fitness clearances were achieved. The antibiotic prophylaxis was carried out in the form of ceftriaxone 1 gram intravenously 15 minutes before the incision during the surgery. The preparation of surgical sites was done within 30 minutes before the procedure and verification of instruments to be used was in advance.

## Technique of operation and instruments:

Each of the patients was treated with dual-plate fixation with two locking compression plates (LCP) of the distal femur. The surgical equipment consisted of anatomically shaped lateral locking compression plates for the distal femur, latter locking compression plates of the proximal tibia adapted to the position of the medial distal femur plate, locking compression cortical screws, variable length, drill bit 4.3mm drill bit 4mm drill bit 2.7mm drill sleeve 5mm and 3.2mm drill sleeve 2mm K-wires screwdrivers; 5mm screwdriver 3.2mm screwdriver pneumatic/battery.

## Surgical:

The approach was lateral and medial approaches to the distal femur, which was plated (on both sides). The L/A technique were to offer simple articular fractures visualization and reduction using an atraumatic split of the vastus lateralis muscle. The medial plate was inserted through medial approach which was used to handle medial femoral condylar injuries. Anesthesia and Patient Positioning: Procedures were carried out using spinal, epidural, or general anesthesia depending on patient condition and the anesthesiologist will use. The patients were placed in supine position and the affected limb was raised by placing the affected limb on the pillow and time was recorded using the pneumatic tourniquet.

## Treatment of open fracture:

The Gustilo-Anderson classification system was used to classify open fractures. A combination of ceftriaxone 1 gram intravenously BID (twice a day) with amikacin 500mg IV BID (twice a day) was introduced as immediate antibiotic therapy in addition to tetanus toxoid prophylaxis. Type I and Type II open Fractures were irrigated with plenty of normal saline, povidone iodine preparation, and temporary immobilisation applied on above knee plaster of Paris up to the time when final fixation could be done. This study excluded type III open fracture.

## Post-operative care and mobilization plan:

Limbs were elevated in Post-operative period; intravenous antibiotics (5-7 days) and oral antibiotics (5 days), the duration of antibiotics is doubled in compound fractures. The administration of drugs to control pain was carried out clinically. On the 14th day of post-op, removal of the suture took place. Mobilization procedures were customized in relation to related soft tissue damages. On the first post-operative day, there was initiation of quadriceps exercising that stands out as being static and active knee and ankle mobilization. Mobilization became non-weight bearing with the first dressing change (48 hours post-operatively), progressing to partial weight bearing following removal of sutures as the pain diminished and full weight bearing 8-10 weeks post-surgery depending on radiographic signs of healing.

## Outcome measurement and follow-up protocol:

Regular follow-ups of patients were maintained at 1, 3, 6, 9 months after surgery, and thereafter every 4 weeks, until the radiographic and functional recovery were achieved. The main outcome measure was the use of the Knee Society Score (KSS) to measure the functional outcomes. The KSS assesses pain and range of motion, stability, and functional capacity based on a scoring system in which an excellent score ranges 80-100, a good score, 70-79, a fair score, 60-69, or poor score is 60 to less. Radiographic healing was an expression indicating full bone remodeling at the area of fracture accompanied by signs of bonding over three cortices in both anteroposterior and lateral radiographic depictions. Also, reduction loss, complications, and total functional outcome were assessed at every follow-up. Follow-up period was between 22 weeks and full union of the fracture was obtained.

## Statistical analysis:

The parametric and non-parametric tests were used to conduct the statistical analysis according to the pattern of data. Demographic factors, surgical factors and outcome measures were subjected to descriptive statistics. Paired t-tests were maintained to compare functional scores obtained pre- and post-operatively. Categorical variables were realized using chi-square test and the relationship of factors to functional outcomes were detected with correlation analysis technique. The statistical significance was determined at p < 0.05 with all the analysis performed on the standard statistical software packages.

## Ethical considerations:

The protocol of the study was agreed within the institutional ethics committee and all practices were according to ethical standards of human research. All the participants signed an informed consent and their confidentiality as patients was taken care of during the period of the study. Any patient could opt out of the study at any point with no implications to his or her clinical treatment.

## Results and Discussion:

In Table 1, the demographic profile demonstrates that distal femur fractures in this series occurred predominantly in young adults, with 60% of patients in the 18-40-year age group and fewer patients in older age categories. This is comparable to the finding of the study done by Shah *et al.* [19] Epidemiological studies show sex- and age-related variation in distal femur fractures, with substantial differences by population and mechanism of injury [20]. The marked male predominance in this cohort (83.3% male vs 16.7% female) is similar to findings reported by Waulden *et al.* [21] and may reflect greater exposure to high-risk behaviours and high-energy trauma including road traffic accidents (RTAs). Right-sided fractures were more common (66.6%), which could be associated with handedness, road systems, or occupation, though this laterality trend needs focused evaluation rather than assumption of causality. As shown in Table 2, RTAs were the major mechanism of injury (76.66%), with falls (20%) and assaults (3.3%) contributing smaller proportions. Several studies identify high-energy mechanisms-especially RTAs in young adults-as major precipitating factors of fractures in this population, particularly among men who have higher exposure to two-wheeler travel and similar risks [22, 23]. Falls were the second most frequent cause, and these injuries can show age- and circumstance-specific patterns (e.g., stairs, standing falls). These distributions are influenced by societal factors such as urbanization, adherence to traffic regulations, and occupational risk [24].

Regarding injury severity, closed fractures comprised 43.33%, while open fractures were common (G.A. type I: 30%; type II: 26.66%). The Gustilo-Anderson classification is widely used for open fractures and has been associated with prognosis and complication risk, with more severe open fractures generally having worse outcomes for infection and other complications [25, 26]. This supports careful soft-tissue management and infection prevention strategies, particularly as open fracture grade increases. Table 3 shows that most procedures (76.66%) took 90-120 minutes, and longer plates (9-11 holes) were used more frequently (66.6%) than shorter plates (6-8 holes, 33.3%). Operative duration is clinically meaningful because longer procedures can increase perioperative exposure and may be associated with higher complication risk in orthopaedic trauma settings, emphasizing the value of efficient technique and strict sterile protocols. In addition, construct design in locked plating influences union; excessive construct stiffness and suboptimal working length/screw density have been associated with healing difficulties in distal femur locked plating, supporting attention to plate length, screw distribution, and "bridge-plating" principles (including leaving holes empty around the fracture zone when appropriate) [27, 28]. Table 4 demonstrates that most fractures united at 20-22 weeks (33.33%), with comparable proportions uniting at 18-20 weeks and 22-24 weeks (each 26.66%). These union times fall within commonly observed healing windows for long-bone fractures and have practical implications for rehabilitation timelines and return to function. Dual plating has been reported to improve union in comminuted distal femur fractures compared with single lateral plating in published cohorts, supporting the biologic and mechanical rationale for adding medial support in complex patterns. Functionally, most patients achieved favorable Knee Society Scores (good 66.66%, excellent 23.33%), while 10% were fair/poor; better functional recovery is typically influenced by factors such as younger age, earlier mobilization, and lower injury complexity, highlighting the importance of rehabilitation in addition to fixation strategy [8]. Table 5 shows complications in 30% of patients, predominantly arthrofibrosis/joint stiffness (16.66%), with smaller proportions of superficial infection, deep infection, and delayed union. Stiffness and delayed union are well-recognized problems after distal femur fracture fixation and are influenced by injury severity, surgical approach, construct characteristics, and postoperative mobilization strategies. Importantly, there were no cases of screw loosening, implant failure, or malunion in this cohort; given that locked plating series have reported nonunion and healing complications even without early hardware failure, the lack of mechanical failure here may reflect adequate construct stability with dual-column support and appropriate postoperative protocols [29].

## Conclusion:

Double distal femur locking compression plate fixation demonstrated superior clinical outcomes compared to single plate fixation in managing comminuted distal femur fractures. The dual plating technique achieved 89.99% excellent to good functional results according to the Knee Society Score with acceptable complication rates of 30%. Despite longer operative duration, dual plating provided enhanced biomechanical stability with faster fracture healing, averaging 20-22 weeks for union. Thus, we show that dual plating is an effective treatment modality for complex comminuted distal femur fractures with satisfactory functional recovery and manageable complications.

## Figures and Tables

**Table 1:** Demographic profile of study participants

**Parameter**	**Number of Patients**	**Percentage (%)**
**Age Distribution**		
18-30 years	9	30%
31-40 years	9	30%
41-50 years	7	23%
51-60 years	5	17%
Total	30	100%
**Sex Distribution**		
Male	25	83.30%
Female	5	16.70%
Total	30	100%
**Side Affected**		
Right	20	66.60%
Left	10	33.30%
Total	30	100%

**Table 2:** Injury and fracture characteristics of study participants

**Parameter**	**Number of Patients**	**Percentage (%)**
**Mode of Injury**		
RTA (Road Traffic Accident)	23	76.66%
Fall	6	20%
Assault	1	3.30%
Total	30	100%
**Fracture Type (Clinical)**		
Closed Fracture	13	43.33%
Open G.A type-I	9	30%
Open G.A type-II	8	26.66%
Total	30	100%
**Fracture Pattern**		
Type C	30	100%
Total	30	100%

**Table 3:** Surgical data of study participants

**Parameter**	**Number of Patients**	**Percentage (%)**
**Duration of Surgery**		
90-120 minutes	23	76.66%
120-150 minutes	7	23.33%
Total	30	100%
**Number of Plate Holes Used**		
6-8 holes	10	33.30%
9-11 holes	20	66.60%
Total	30	100%

**Table 4:** Fracture union and outcome

**Parameter**	**Number of Patients**	**Percentage (%)**
**Duration of Fracture Union (Weeks)**		
16-18 weeks	0	0%
>18-20 weeks	8	26.66%
>20-22 weeks	10	33.33%
>22-24 weeks	8	26.66%
>24-26 weeks	2	6.66%
>26-28 weeks	2	6.66%
Total	30	100%
**Results (Knee Society Score)**		
Excellent	7	23.33%
Good	20	66.66%
Fair	2	6.66%
Poor	1	3.33%
Total	30	100%

**Table 5:** Post surgery complications

**Complication**	**Number of Patients**	**Percentage (%)**
Superficial Skin Infection	1	3.33%
Arthrofibrosis (Joint Stiffness)	5	16.66%
Deep Infection	1	3.33%
Delayed Union	2	6.66%
Screw Loosening	0	0%
Implant Failure	0	0%
Malunion	0	0%
Total	9	30%
